# P53/miR-34a/SIRT1 positive feedback loop regulates the termination of liver regeneration

**DOI:** 10.18632/aging.203920

**Published:** 2023-03-28

**Authors:** Junhua Gong, Minghua Cong, Hao Wu, Menghao Wang, He Bai, Jingyuan Wang, Keting Que, Kaiwen Zheng, Wenfeng Zhang, Xiaoli Yang, Jianping Gong, Hanping Shi, Mingyong Miao, Fangchao Yuan

**Affiliations:** 1 Department of Hepatobiliary Surgery, The Second Affiliated Hospital of Chongqing Medical University, Chongqing 400000, China; 2 Comprehensive Oncology Department, National Cancer Center/National Clinical Research Center for Cancer/Chinese Academy of Medical Sciences and Peking Union Medical College, Cancer hospital, Beijing 100021, Beijing, China; 3 Department of General Surgery, The First Affiliated Hospital of Xi’an Medical University, Lianhu, Xi’an 710000, Shaanxi Province, China; 4 Department of Orthopaetics, Dianjiang People’s Hospital of Chongqing, Chongqing 408300, Chongqing, China; 5 Department of Hepatobiliary Surgery, The Affiliated Hospital of Southwest Medical University, Luzhou 646000, Sichuan Province, China; 6 Department of Gastrointestinal Surgery/Clinical Nutrition, Beijing Shijitan Hospital, Capital Medical University, Beijing 100038, China; 7 Institute of BioPharmaceutical Research, Liaocheng University, Liaocheng 252059, China

**Keywords:** liver regeneration, HCC, P53, miR-34a, SIRT1

## Abstract

Background: The capacity of the liver to restore its architecture and function assures good prognoses of patients who suffer serious hepatic injury, cancer resection, or living donor liver transplantation. Only a few studies have shed light on the mechanisms involved in the termination stage of LR. Here, we attempt to further verify the role of the p53/miR-34a/SIRT1 positive feedback loop in the termination of liver regeneration and its possible relationship with liver cancer.

Method: We performed partial hepatectomy (PH) in mice transfected with adenovirus (Ade) overexpressing P53 and adenovirus-associated virus (AAV) overexpressing miR-34a. LR was analyzed by liver weight/body weight, serum alanine aminotransferase (ALT) and aspartate aminotransferase (AST) levels and cell proliferation, and the related cellular signals were investigated. Bile acid (BA) levels during LR were analyzed by metabolomics of bile acids.

Results: We found that the P53/miR-34a/SIRT1 positive feedback loop was activated in the late phase of LR. Overexpression of P53 or miR-34a terminated LR early and enhanced P53/miR-34a/SIRT1 positive feedback loop expression and its proapoptotic effect. T-β-MCA increased gradually during LR and peaked at 7 days after PH. T-β-MCA inhibited cell proliferation and promoted cell apoptosis via facilitating the P53/miR-34a/SIRT1 positive feedback loop during LR by suppressing FXR/SHP. The P53/miR-34a/SIRT1 positive feedback loop was abolished in HCC patients with P53 mutations.

Conclusions: The P53/miR-34a/SIRT1 positive feedback loop plays an important role in the termination of LR. Our findings showed the molecular and metabolic mechanisms of LR termination and provide a potential therapeutic alternative for treating P53-wild-type HCC patients.

## INTRODUCTION

The astonishing regenerative capacity of the liver has been studied by scientists for many years. The incredible capacity of the liver to regain its architecture and function assures good prognoses of patients who suffer liver cancer resection, severe injury of liver or living donor liver transplantation [1]. Thus, a better understanding of the mechanism involved in LR helps us gain insights into the cause of many kinds of acute and chronic liver diseases and peculiarly on hepatocarcinogenesis. The complete process of LR, which consists of three stages including initiation, proliferation and termination part, requires numerous growth factors and cytokines, such as HGF, TNF-α, EGF, and TGF-β [2].

Although researchers have studied LR for decades, most of their work has focused on the initiation stage of LR, while mechanism involved in the termination phase of LR remain to be elucidated. Only a few studies have shed light on the mechanisms underlying the termination stage of LR. It has been reported that TGF-β originate from hepatocytes and nonparenchymal cells in liver could suppress DNA synthesis in regenerating hepatocytes [3]. Other researchers have proven that IL-1a is another inhibitor of liver regeneration and suppresses DNA synthesis in hepatocytes [4]. A recent study reported that PP2Acα terminates LR in mice via the AKT/GSK3β/Cyclin D1 signaling pathway [5]. Ian Huck found that knockout of hepatocyte nuclear factor 4 alpha increased hepatocyte proliferation throughout LR and that HNF4a activation was crucial for terminating liver regeneration in mice [6].

A previous study by Miao confirmed that miR-34a was elevated in the termination phase of LR in rats and suppressed the proliferation of hepatocytes during LR [7]. Furthermore, miR-34a-induced cell death might be related to the cellular context and the miRNA targets, which promote apoptotic cell death. miR-34a has been reported to be one of the direct target gene of P53 [8]. Sirtuin 1 (Sirt1), which is a target gene of miR-34a, regulates cell apoptosis. P53 acetylation and transcriptional activation induced by miR-34a via Sirt1 suppression eventually leads to apoptosis. Thus, P53, miR-34a and SIRT1 formed a positive feedback loop, in which P53 activates miR-34a, while miR-34a induces acetylation and transcription of P53 by repressing SIRT1 to promote cell apoptosis [9, 10]. Theoretically, if not controlled, the proapoptotic effect of the P53/miR-34a/SIRT1 positive feedback loop would be reinforced infinitely. Jiyoung Lee et al. proved that the activation of miR-34a by P53 can be inhibited by the FXR/SHP signaling pathway [11]. FXR has been long known as a receptor of bile acids (BAs). Therefore, we intend to explore to what extent the P53/miR-34a/SIRT1 positive feedback loop is involved in the termination of LR and whether there is a putative BA functioning as a regulatory factor of the P53/miR-34a/SIRT1 pathway through the FXR/SHP signaling pathway. Moreover, unlike the uncontrollable proliferation of liver cancer cells, normal liver regeneration can be terminated in time, and the size of the liver can be accurately controlled. Therefore, the disorder of the mechanism involved in the termination of liver regeneration may be linked to the onset and progression of liver cancer. We also explored the relationship between the p53/miR-34a/SIRT1 positive feedback loop and HCC.

## MATERIALS AND METHODS

### Patients and sample collection

We collected HCC tissues and their paired adjacent tissues (5 cm from tumor edge) from patients who had liver tumor resected at the Department of Hepatobiliary Surgery from The Second Affiliated Hospital of Chongqing Medical University between March 2013 and December 2015. None of the patient undergone any radiotherapy or chemotherapy prior to radical or partial hepatectomy. All patients enrolled in this study signed informed consent form and the whole process was certified and supervised by the Ethic Committee of The Second Affiliated Hospital of Chongqing Medical University.

All samples were put into liquid nitrogen for snap-frozen as soon as possible and stored at − 80° C until use.

### Experimental animals

Experiments were carried out using C57BL/6 mice purchased from Experimental Animal Center affiliated to Chongqing Medical University (male, weight varying from 19 to 22 g). (Chongqing, China). Prior to the experiments, standard chow and water were given to the mice ad libitum. All mice were kept in cages in a facility in a12-hour light/12-hour dark cycle under a constant temperature of 23° C and humidity of 60%. The animal experiments were approved by the China Association of Laboratory Animal Care, and we made all we could to minimize the mice’s suffering.

### Adenovirus

Adenovirus (Ade) expressing green fluorescent protein (GFP) and a sequence targeting P53 was constructed by ABM (Nanjing, China). Ade-GFP was used as a control. Mice were given tail vein injections of adenovirus at the concentration of 5 × 10^11^ genome-equivalents five days before surgery.

### Adeno-associated virus-8

Adeno-associated virus-8 (AAV8) carrying GFP and anti-miR-34a sequence was design and constructed by ABM (Nanjing, China). Mice were given tail vein injections of AAV8 at the concentration of 5 × 10^11^ genome-equivalents two weeks before surgery. AAV8-GFP was used as control.

### Partial hepatectomy

Mice were divided into the following groups at random: Ade-GFP group: mice transfected with adenovirus carrying GFP. Ade-p53 group: mice transfected with adenovirus carrying P53; AAV8-GFP: mice transfected with AAV8 carrying GFP; AAV-anti-miR-34a group: mice transfected with AAV8 knockdown of miR-34a. Mice ether received sham or PH surgery (sham, n = 9; PH, n = 9 per time point).

### MCA experiment

(1) Normal diet group (ND): mice in this group received only normal diet (2) T-β-MCA group: mice in this group received normal diet and gavage with extra T-β-MCA (400 mg/kg, Steraloids, Cat# C1899-000) 1 day before PH and every 3 days after PH. Mice were subjected to traditional PH as previously reported [2]. Briefly, Sodium pentobarbital (50 mg/kg) was administered intraperitoneally to anesthetize the mice.

At specific times (0, 2, 3, 5, 7, 10, 14 days) after surgery, mice were euthanized by inhaling overdose (2% to 3% isoflurane) of anesthetic, and we took whole blood of mice through puncture in portal vein. Before being dissected and weighed, we perfused liver tissues as soon as possible with ice-cold PBS.

The following equation was used to calculate the liver/body weight ratio: liver/body weight ratio = (remnant liver weight [g]/body weight [g]) × 100%.

### Cell isolation and purification

As previously stated, primary hepatocytes were extracted using the collagenase perfusion method [12]. In short, mice were thoroughly anesthetized and disinfected, while the liver was promptly injected with buffers 1 and 2 before being removed and moved to a cell culture plate with a diameter of 100 mm prepared for mechanical dissociation. The suspension of liver cells was distributed into a 12-well culture plates and incubated at 37° C in a 5% CO2 environment.

### Cell transfection

Primary mouse hepatocytes were transfected with adenovirus particles with knock-in of wild-type P53. For the control, the cells hepatocytes were transfected by NC or ade-GFP. All primers were synthetized by ABM (Nanjing, China). The cells were collected for further study.

### Measurement of bile acid metabolites

We first suspended liver tissue in sodium hydroxide at a concentration of 1 M, and then resuspended them in methanol and pyridine. Then, to begin derivatization, we added MCF into it and blended it via vortexing. Next, we added chloroform and bicarbonate at a concentration of 50 mM and blended it. Finally, centrifugation (12000×g, 5 min, 24° C) was used to get the organic layers of the samples. After adding anhydrous sodium sulfate, we put samples which have been derivatized into the glass inserts piece inside the liquid chromatography (LC) vials, while we treated empty tubes with the same process used as negative controls. To measure the differential expression of metabolites, the derivatized extracts were examined with an ultrahigh-performance liquid chromatography (UHPLC-MS/MS) equipment [13].

### Liver histopathology

Liver tissues, which were preserved in neutral formalin (10%), were then fixed in paraffin and cut into sections which were 5 μm thick. After baked at 60° C for 1 h, the sections were dehydrated in gradient ethanol and xylene. Next, we stained the sections in eosin for 30 min. We rinsed the sections briefly by running water, again the sections were dehydrated and sealed in neutral gum.

### Detection of liver function

The blood samples from mice received PH at different time points were centrifuged for 5 min at a speed of 3000 r/min to collect the upper translucent serum layer. Serum alanine aminotransferase (ALT) and aspartate aminotransferase (AST) levels were tested in the clinical biochemical laboratory at the relevant times using a standard automatic biochemistry analyzer.

### Immunohistochemical evaluation

Liver tissues, which were preserved in neutral formalin (10%), were then embedded in paraffin and cut into sections which were 5 μm thick [14]. The primary antibodies used: P53 (cat. No. Ab26, Abcam Inc.) and PCNA (cat. No. Ab92552, Abcam Inc.) at a concentration of 1:50. At room temperature, the secondary streptavidin-horseradish peroxidase-conjugated antibody staining was carried out. After the sections were dehydrated, they were sealed by using neutral resin. The specific staining was observed under light microscopy [15].

### RNA isolation and quantification

TRIzol kit and PrimeScript™ 1st Strand cDNA synthesis kit from Takara (Otsu, Japan) were used for the isolation of total RNA from liver tissues and then reverse-transcription from RNA to cDNA. The primer of miR-34a and internal reference U6 and the miDETECT A Track miRNA qRT-PCR Starter Kit for performing qRCR were purchased from Guangzhou RiboBio Co., LTD (Guangzhou, China).

Applied Biosystems 7500 Real-time PCR (Thermo Fisher Scientific Inc.) was used to performing qPCR. The relative expression of genes was calculated using the 2^-∆∆Ct^ method [16].

### Western blot analysis

Proteins were extracted with 1× RIPA buffer (Beyotime, Shanghai, China) and denatured after heating at a temperature of 100° C for 10 mins. Forty micrograms of protein were added to each lane, separated on a 10% SDS/PAGE gel by electrophoresis and then the targeted proteins were electrotransferred onto PVDF membranes. After we blocked the membranes by 5% nonfat milk for 1 h, they were incubated primary antibodies dilutions at the temperature of 4° C overnight. Primary antibodies against the following antigens were used and showed in Supplementary Table 2.

P53 (cat. No. Ab26; 1:1000; Abcam Inc.), Ace-P53 (cat. No. 2570S; 1:1000; CST.), SIRT1 (cat. No. 8469S; 1:1000; CST), cleaved Caspase3 (cat. No. Ab231289; 1:1000; Abcam Inc.), P21 (cat. No. Ab188224; 1:1000; Abcam Inc.), Bax (cat. No. Ab32503; 1:1000; Abcam Inc.), NR1H4 (FXR) (cat. No. Ab187735; 1:1000; Abcam Inc.), and NR0B2 (SHP) (cat. No. Ab186874; 1:1000; Abcam Inc.). The membranes were then incubated with species-matched secondary antibodies. After incubated in the species-matched secondary antibodies and washed by PBS, the protein bands on membranes were exposed and recorded by the Bio-Rad ChemiDoc XRS system (Hercules, CA, USA). We analyzed the images by ImageJ software.

### TUNEL apoptosis assays

A one-step TUNEL assay kit-green fluorescein (Cat. No. C1086, Beyotime Biotechnology Inc.) was used to detect apoptosis. First, liver sections underwent deparaffinizing and dehydrating. After washed by the wash buffer (0.1% Triton X-100 in PBS) for 5 min, the sections were incubated by 50 μl of TUNEL reaction mixture in the dark at 37° C for 1 h, and then DAPI was used for counterstaining. Slides were mounted and observed under an immunofluorescence microscope after being washed.

### Statistical analysis

SPSS 22.0 software was used to process the statistical analyses and all values are expressed as the mean±SD. Statistical significance of differences was calculated using t-tests for parametric data involving two groups and one-way analysis of variance with Tukey’s test for multiple groups. We used ANOVA repeatedly to compare datasets at different time points. We used log-rank test to compare the differences between the survival curves. Differences were considered statistically significant when *P*<0.05.

### Availability of data and materials

The data sets used and/or analyzed during the current study are available from the corresponding author on reasonable request.

### Consent for publication

The complete clinical and prognostic data for each tumor tissue sample were recorded, and human tumor tissues used for this research were obtained with informed consent. The study was conducted in accordance with the protocol approved by the Declaration of Helsinki and the guidelines of the Ethics Review Committee of the second Affiliated Hospital of Chongqing Medical University.

## RESULTS

### The P53/miR-34a/SIRT1 proapoptotic pathway is activated in the termination stage of LR

**Figure 1:**
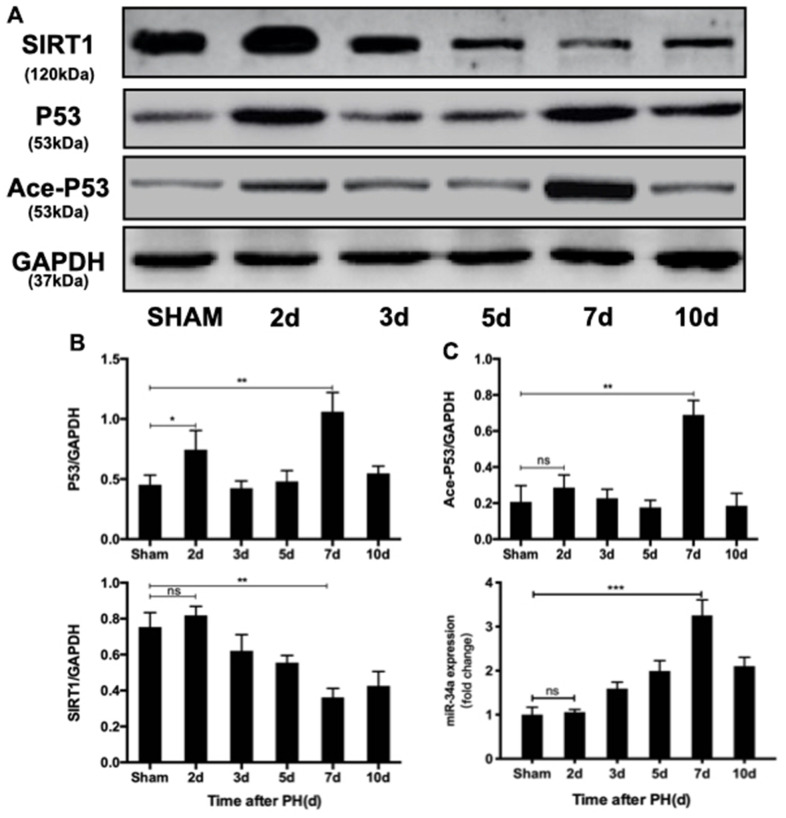
**P53/miR-34a/SIRT1 positive feedback loop expression during LR.** (**A**) Protein expression of the P53/miR-34a/SIRT1 positive feedback loop during LR progression was analyzed via western blot. (**B**) Quantification of hepatic P53, Ace-P53, and SIRT1 expression at the indicated time points after PH by WB. (**C**) Quantification of hepatic miR-34a expression at the indicated time points after PH by qPCR. (ns: not significant, p>0.05; *, p<0.05; **, p<0.01; ***, p<0.001).

To investigate the role of P53/miR-34a/SIRT1 positive feedback loop during LR, first we discovered whether P53/miR-34a/SIRT1 loop was activated after PH. P53 remarkably increased at both day 2 and day 7 after PH, while no significant change was detected in miR-34a at day 2 of PH (Figure 1A, 1C). Thus, enhanced P53 level failed to activate P53/miR-34a/SIRT1 loop at day 2 after PH. The level of acetylated P53 was in accordance with miR-34a expression. SIRT1 decreased gradually from day 2 to day 7 after PH and increased thereafter (Figure 1A, 1C). As LR progressed, miR-34a expression gradually increased, peaked at day 7 after PH and decreased sharply thereafter (Figure 1B). Therefore, our data showed that the P53/miR-34a/SIRT1 loop is activated in the late stage of PH, which might stimulate LR termination.

### Overexpression of wild-type P53 terminates LR and enhanced the P53/miR-34a/SIRT1 positive feedback loop during LR *in vivo*

**Figure 2:**
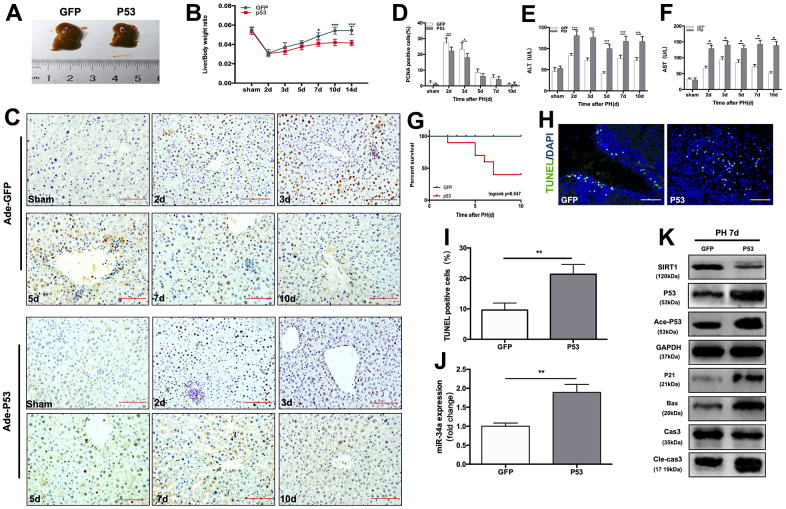
**Overexpression of wild-type P53 terminates LR and activates the P53/miR-34a/SIRT1 positive feedback loop early during LR.** (**A**) Representative livers from mice at 7 days after PH between Ade-GFP and Ade-P53 mice. (**B**) Liver weight relative to body weight at the indicated time points after PH. (**C**) Representative images of PCNA staining at the indicated time points after PH from the Ade-GFP and Ade-P53 groups (magnification: ×200, scale bars represent 50 μm). (**D**) Quantification of PCNA-positive cells in the liver at the indicated time points after PH. (**E**, **F**) Serum AST and ALT levels in Ade-GFP and Ade-P53 mice after PH. (**G**) Survival rate of mice that underwent PH from the Ade-GFP and Ade-P53 groups(p=0.047, logrank test). (**H**) Representative images of TUNEL staining at day 7 after PH in liver tissue (magnification: ×400). (**I**) Quantification of TUNEL-positive cells in the liver at day 7 after PH. (**J**) Quantification of hepatic miR-34a expression between Ade-GFP and Ade-P53 mice at day 7 after PH by qPCR. (**K**) Protein expression of P53/miR-34a/SIRT1 positive feedback loop genes between Ade-GFP and Ade-P53 mice day 7 after PH. (*, p<0.05; **, p<0.01; ***, p<0.001).

In order to further study the role of the P53/miR-34a/SIRT1 loop in LR, we performed PH on mice that received tail-vein injection of adenovirus overexpressing P53 (Supplementary Figure 1). When compared to the size of regenerated livers in mice from the Ade-GFP group, mice from the Ade-P53 group had significantly smaller regenerated liver size (Figure 2A). The liver-to-body weight ratio at day 7, day 10 and day 14 was significantly lower in the Ade-P53 group, and the liver-to-body weight ratio stopped increasing starting on day 5 in the Ade-P53 group (Figure 2B). Then, we compared the proliferation of cells in the liver during LR, and we observed more PCNA-positive cells in the Ade-GFP group on day 2 and 3 after PH (Figure 2C, 2D). The levels of ALT and AST in the Ade-GFP group spiked soon after surgery, then steadily reduced as the liver mass and physiological structures started to recover. In Ade-P53 mice, however, the serum levels of ALT and AST were considerably higher and did not return to normal when compared to GFP mice (Figure 2E, 2F). The survival rate of mice that received PH surgery decreased sharply to 50% in the Ade-P53 group (Figure 2G). TUNEL-positive cells in liver increased in P53 group (Figure 2H, 2I). When P53 was overexpressed, the P53/miR-34a/SIRT1 loop expression was enhanced at 7 days after PH (Figure 2J, 2K). Therefore, these data suggest that knock-in of P53 enhanced P53/miR-34a/SIRT1 positive feedback loop during LR and terminates LR early.

### Knock-down of miR-34a inhibited the P53/miR-34a/SIRT1 positive feedback loop during LR and postpones LR termination *in vivo*

**Figure 3:**
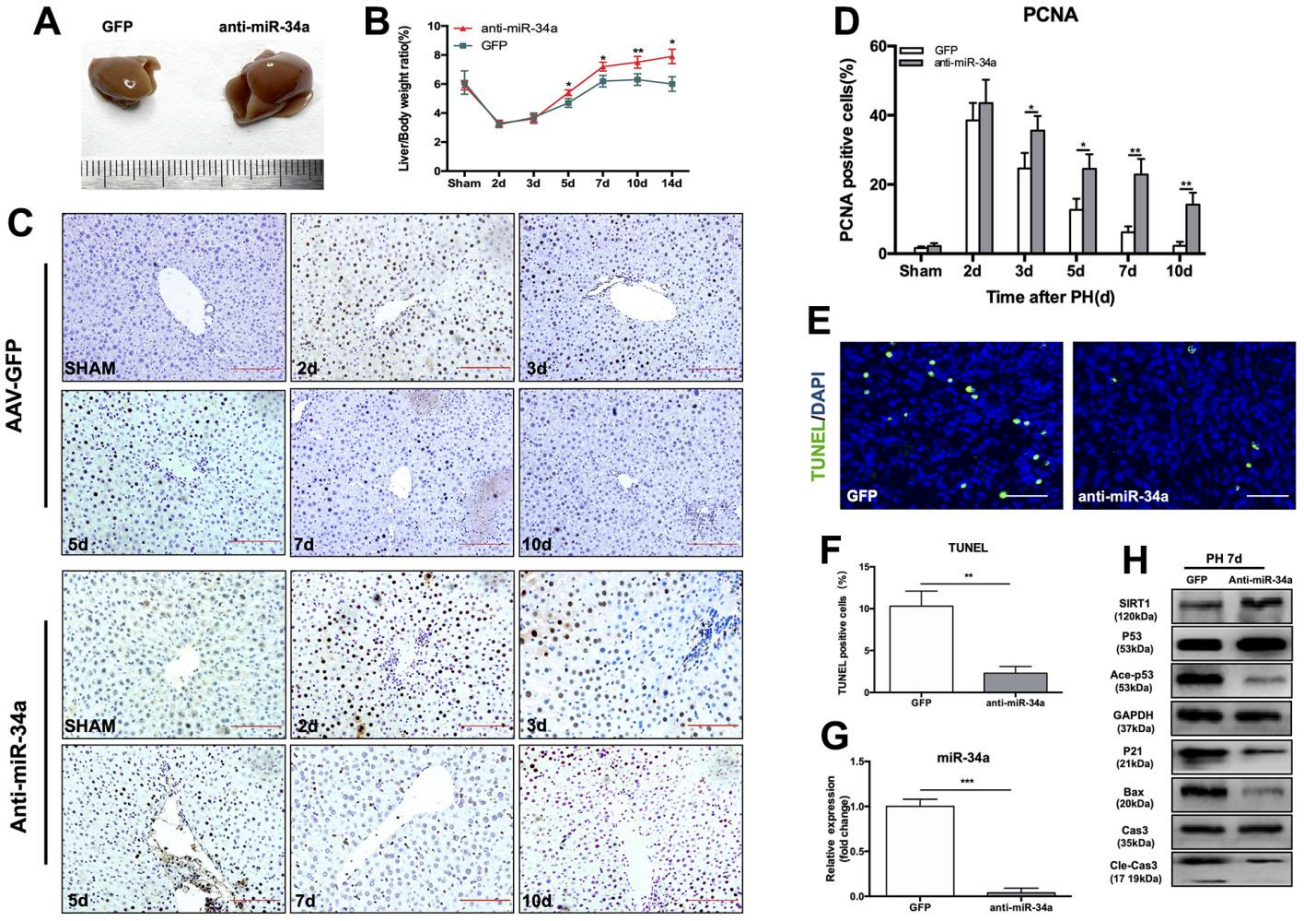
**Knock-down of miR-34a suppresses the P53/miR-34a/SIRT1 positive feedback loop early during LR.** (**A**) Representative livers from mice at 7 days after PH between AAV-GFP and AAV-anti-miR-34a mice. (**B**) Liver weight relative to body weight at the indicated time points after PH. (**C**) Representative images of PCNA staining at the indicated time points after PH in the GFP and anti-miR-34a groups (magnification: ×200, scale bars represent 50 μm). (**D**) Quantification of PCNA-positive cells in the liver at the indicated time points after PH. (**E**) Representative images of TUNEL staining at day 7 after PH in liver tissue (magnification: ×400). (**F**) Quantification of TUNEL-positive cells in the liver at day 7 after PH. (**G**) Protein expression of P53/miR-34a/SIRT1 positive feedback loop genes between AAV-GFP and AAV-miR-34a mice at day 7 after PH. (**H**) Quantification of hepatic miR-34a expression between AAV-GFP and AAV-miR-34a mice at day 7 after PH by qPCR. (*, p<0.05; **, p<0.01; ***, p<0.001).

In order to further study the function of the P53/miR-34a/SIRT1 loop in LR, we performed PH on mice that received tail-vein injection of AAV that down-regulated miR-34a (Supplementary Figure 2). At day 7 after PH, mice in the anti-miR-34a group had substantially larger regenerated livers than mice in the AAV-GFP group (Figure 3A). The liver-to-body weight ratio at 5, 7, 10 and 14 days after PH were significantly higher in the anti-miR-34a group (Figure 3B). We next assessed cell proliferation by PCNA staining, and cell proliferation in the liver was promoted in the anti-miR-34a group at 3, 5, 7, 10 days after PH (Figure 3C, 3D). TUNEL-positive cells in liver decreased sharply in anti-miR-34a group (Figure 3E, 3F). When miR-34a was knocked down, the genes in P53/miR-34a/SIRT1 loop was down-regulated, as were the proapoptotic genes downstream (Figure 3G, 3H). Therefore, these data suggest that knock-down of miR-34a broke the P53/miR-34a/SIRT1 loop during LR and LR could not be terminated then.

### T-β-MCA enhanced the proapoptotic effect of the P53-activated P53/miR-34a/SIRT1 positive feedback loop by suppressing the FXR/SHP signaling pathway *in vitro*

It has been proven that BAs participate in LR by acting as signaling molecules that activate signaling pathways [17, 18]. Previous studies have confirmed a stimulatory role of BA at physiological concentrations in LR [19, 20]. Here, we analyzed BAs in the regenerated liver tissue of mice. In our study, we found that there was a difference in the activation of the P53/miR-34a/SIRT1 positive feedback loop between the early and late stages of LR (Figure 1A). P53 level increased at both 2 days and 7 days after PH (Figure 1A). The miR-34a level did not increased, nether the P53/miR-34a/SIRT1 positive feedback loop were activated at 2 days after PH (Figure 1A, 1B). Therefore, P53 transactivation of miR-34a was inhibited in the early stage of LR. We postulated that this might be due to the change in the BA pool during LR.

**Figure 4:**
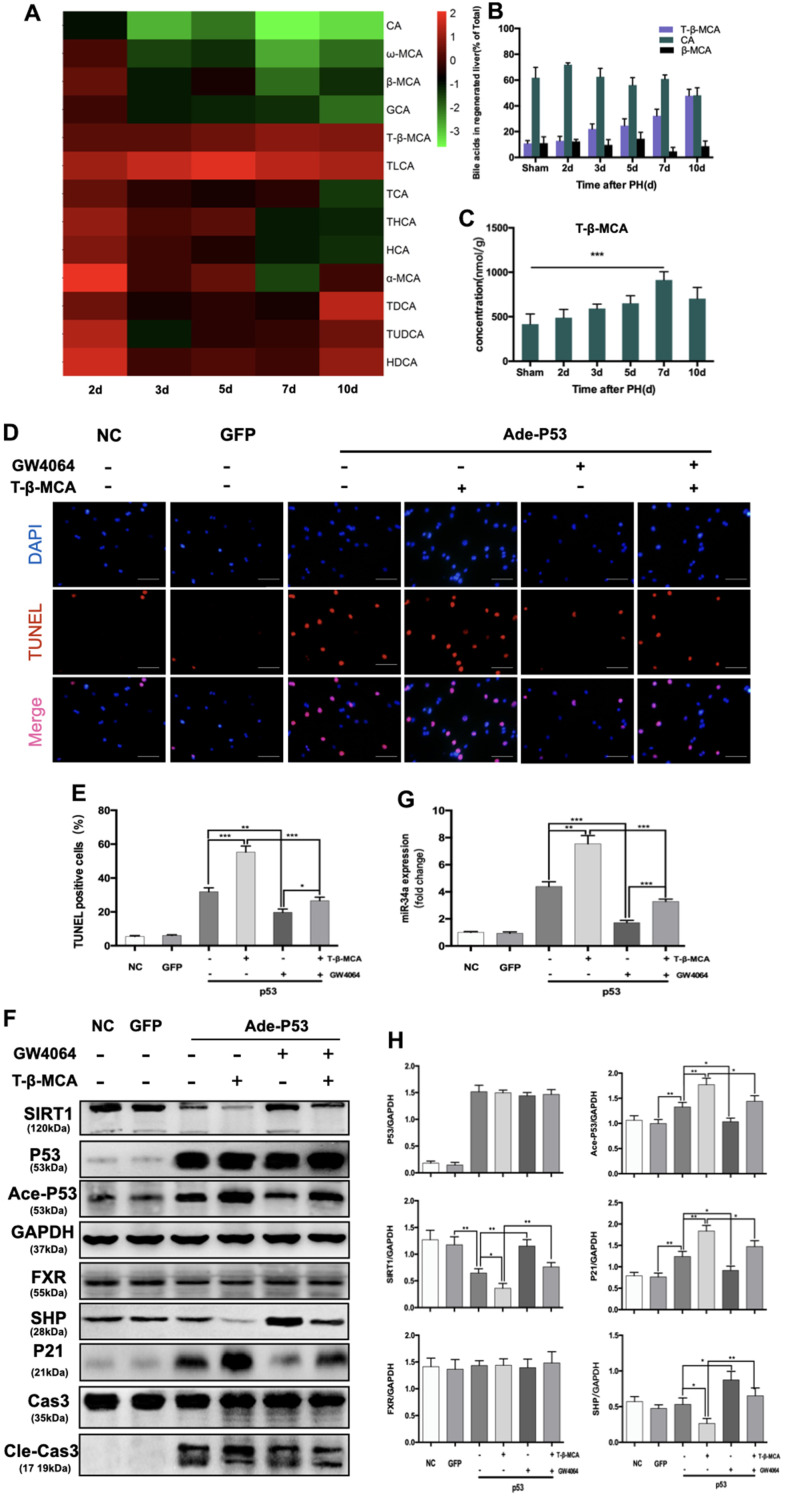
**T-β-MCA enhanced the proapoptotic effect of the P53-activated P53/miR-34a/SIRT1 positive feedback loop by suppressing the FXR/SHP signaling pathway *in vitro*.** (**A**) Heatmap of changed Bas during LR. (**B**) Percentage of the top 3 BAs at the indicated time points after PH. (**C**) Concentration of T-β-MCA at the indicated time points after PH. (**D**) TUNEL staining of primary hepatocytes after knock-in of P53 with/without administration of T-β-MCA and GW4064(magnification: × 400, Scale bars represent 50 μm). (**E**) Quantification of TUNEL-positive cells after knock-in of P53 with/without administration of T-β-MCA and GW4064. (**F**) Quantification of hepatic miR-34a expression in mouse primary hepatocytes after knock-in of P53 with/without administration of T-β-MCA and GW4064 by qPCR. (**G**) Protein expression of P53/miR-34a/SIRT1 positive feedback loop genes and FXR/SHP signaling after knock-in of P53 with/without administration of T-β-MCA and GW4064. Cells were pretreated with/without 100 μM T-β-MCA or 1 μM GW4064 for 12 h. (**H**) Quantification of P53, Ace-P53, SIRT1, P21, FXR, and SHP protein expression by WB. (ns: p>0.05; *, p<0.05; **, p<0.01; ***, p<0.001).

We performed metabolomics of BAs during LR, which included 22 BAs in total, by UHPLC (Supplementary Table 1). Among these BAs, 13 changed significantly during LR in our study (Figure 4A). The levels of 5 BAs were elevated during LR. The levels of 8 BAs decreased in the latter phase of LR. Among the top 3 BAs with the highest concentrations, T-β-MCA increased gradually during LR (Figure 4B). We observed that T-β-MCA level was correlated with the level of miR-34a. As LR progressed, T-β-MCA level in liver gradually grew, peaked at day 7 after PH and decreased sharply thereafter (Figure 4C). Therefore, these data suggest that the P53/miR-34a/SIRT1 positive feedback loop might be activated by T-β-MCA.

Beta-muricholic acid (bMCA) is a major BA in rats and mice [21]. In a recent major study, Fredrik Backhed and his colleagues proved that T-β-MCA is a competitive and reversible FXR antagonist [22]. The transcriptional activation of P53 on miR-34a was inhibited by activated FXR/SHP signaling pathway [11]. Therefore, it is reasonable for us to hypothesize that T-β-MCA is able to suppress FXR/SHP signaling pathway to enhance the effect of the P53-activated P53/miR-34a/SIRT1 positive feedback loop.

We transfected primary mouse hepatocytes with adenovirus carrying wild-type P53. The transfection efficiency was verified by WB and fluorescent imaging (Supplementary Figure 3). In our study, TUNEL staining showed that the apoptosis of hepatocytes was enhanced by T-β-MCA (Figure 4D, 4E). We found that P53 overexpression activated the P53/miR-34a/SIRT1 positive feedback loop in primary mouse hepatocytes, while it was enhanced by the administration of T-β-MCA downstream of proapoptotic genes (Figure 4G, 4H). The FXR/SHP signaling pathway is inhibited by T-β-MCA treatment. Treatment with GW4064, an FXR/SHP signaling pathway agonist, was able to activate the FXR/SHP signaling pathway and inhibit the P53/miR-34a/SIRT1 positive feedback loop in primary mouse hepatocytes. When cells were co-incubated with T-β-MCA, the GW4064-induced activation of the FXR/SHP signaling pathway was inhibited.

### T-β-MCA suppresses facilitates the P53/miR-34a/SIRT1 positive feedback loop during LR by inhibition of FXR/SHP pathway in mice

**Figure 5:**
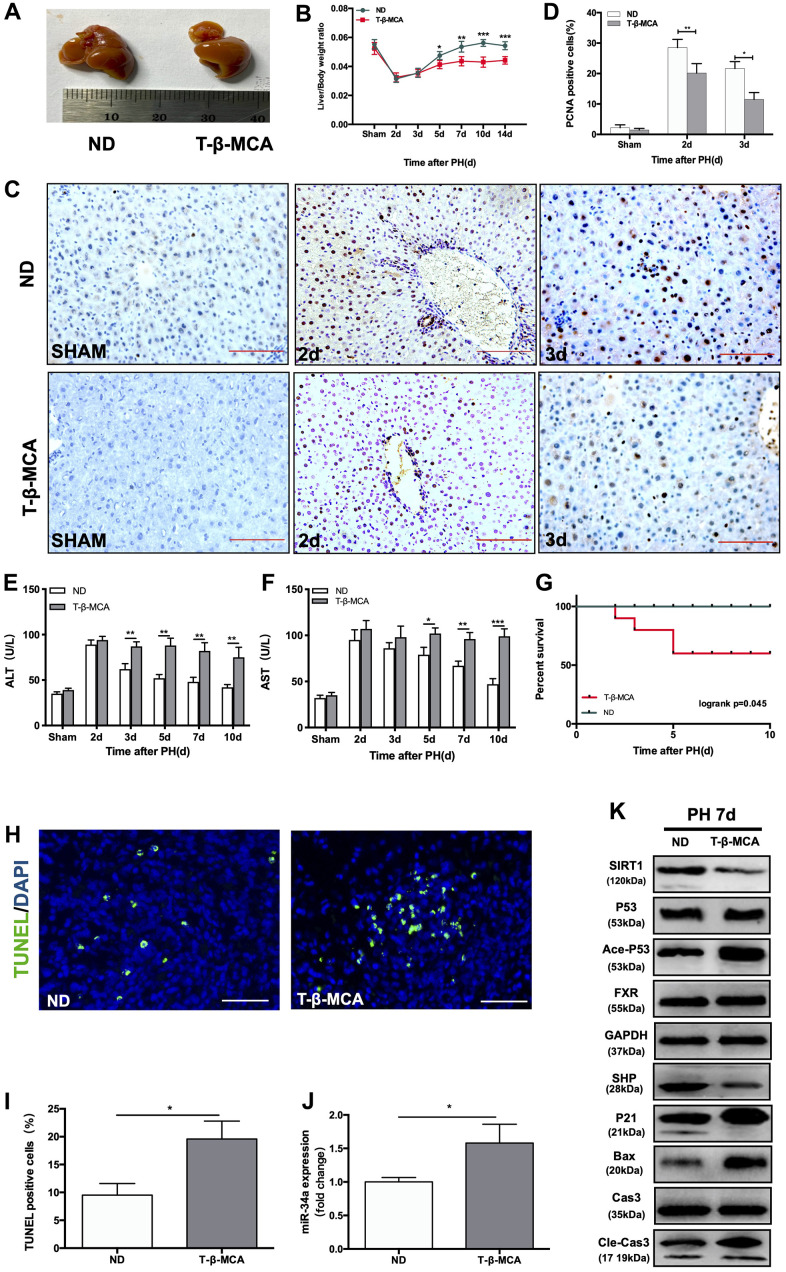
**T-β-MCA facilitates the P53/miR-34a/SIRT1 positive feedback loop during LR by suppressing the FXR/SHP signaling pathway *in vivo*.** (**A**) Representative livers from mice at 7 days after PH between ND and T-β-MCA mice. Mice in the T-β-MCA group were administered T-β-MCA (400 mg/kg) by gavage 1 day before PH and every 3 days after PH. (**B**) Liver weight relative to body weight at the indicated time points after PH. (**C**) Representative images of PCNA staining at the indicated time points after PH from the ND and T-β-MCA groups. (magnification: × 200, Scale bars represent 50 μm). (**D**) Quantification of PCNA-positive cells in the liver at the indicated time points after PH. (**E**, **F**) Serum AST and ALT levels in mice from the ND and MCA groups after PH. (**G**) Survival rate of mice that underwent PH from the ND and T-β-MCA groups(p=0.045, logrank test). (**H**) Representative images of TUNEL staining at day 7 after PH in liver tissue (magnification: ×400). (**I**) Quantification of TUNEL-positive cells in the liver at day 7 after PH. (**J**) Quantification of hepatic miR-34a expression in mice with/without administration of T-β-MCA. (**K**) Protein expression of P53/miR-34a/SIRT1 positive feedback loop genes and FXR/SHP signaling with/without administration of T-β-MCA. *, p<0.05. (ns: not significant, p>0.05; *, p<0.05; **, p<0.01; ***, p<0.001).

We next performed PH on mice fed a normal diet (ND) and mice fed extra T-β-MCA in sterile water by gavage. At 7 days after PH, the size of regenerated livers in the T-β-MCA group was considerably smaller than that in ND group. (Figure 5A). The liver-to-body weight ratio at days 5, 7, 10 and 14 after PH was significantly lower in the T-β-MCA group (Figure 5B). PCNA staining showed that cell proliferation was suppressed in the T-β-MCA group (Figure 5C, 5D). However, the levels of ALT and AST in mice serum were significantly higher and did not recover to normal in T-β-MCA mice compared with ND mice (Figure 5E, 5F). The survival rate of mice that received PH surgery decreased sharply to 50% in the Ade-P53 group (Figure 5G). TUNEL-positive cells in liver increased in T-β-MCA group (Figure 5H, 5I). WB showed that the effect of the P53/miR-34a/SIRT1 positive feedback loop increased, while FXR/SHP was inhibited in the T-β-MCA group (Figure 5J, 5K). Therefore, these data suggest that T-β-MCA is able to facilitate the proapoptotic effect of the P53-activated P53/miR-34a/SIRT1 positive feedback loop by suppression of the FXR/SHP pathway and terminate LR early *in vivo*.

### The expression of the P53/miR-34a/SIRT1 positive feedback loop in hepatocellular carcinoma patients

Hepatocellular carcinoma (HCC), which was deemed as a malignant tumor, is the third leading cause of cancer-related death [23]. Both LR and HCC are characterized by high cell proliferation, but there are significant differences between the two. The unlimited proliferation of HCC cells lacks a termination mechanism, while during LR, cell proliferation can be terminated, and liver mass is precisely regulated. Due to TP53 mutations, the P53 response pathway is frequently deficient in HCC patients [24, 25]. Thus, we hypothesized that P53/miR-34a/SIRT1 fails to function during HCC due to the loss or mutation of P53.

First, we divided 92 HCC patients based on P53-IHC staining into P53-mutated and P53-undetected groups. If the IHC of P53 was positive, the patient was classified as P53-mutated (P53-mut). Then, we divided the P53-negative patients classified by IHC into P53-deficient and P53-wild-type (P53-wt) through WB (Figure 6A). If there was a band in WB, the patient was classified in the P53-wild-type group (P53-wt). If there were no bands in WB, the patient was classified in the P53-deficient group (P53-de). The patient data are shown in (Table 1). We compared the expression of related gene in P53/miR-34a/SIRT1 positive feedback loop in liver cancer tissue(C) and adjacent normal liver tissue(N). When compared with P53-wt patients, P53-mut and P53-de patients has larger tumor size, and P53-mut and P53-de patients are more likely to have distant metastasis. We found that the 5-year survival of P53-deficient (P53-de) patients was the worst among patients from all 3 groups (Figure 6B). In P53-mut patients, IHC staining of P53 was positive, while the P53/miR-34a/SIRT1 positive feedback loop was absent in tumor tissue (Figure 6C–6E). In P53-de patients, IHC staining of P53 was negative, and the P53/miR-34a/SIRT1 positive feedback loop was absent in tumor tissue as well (Figure 6F–6H). In P53-wt patients, IHC staining of P53 was negative as well, while the P53/miR-34a/SIRT1 positive feedback loop was activated in tumor tissue (Figure 6I–6K).

**Figure 6:**
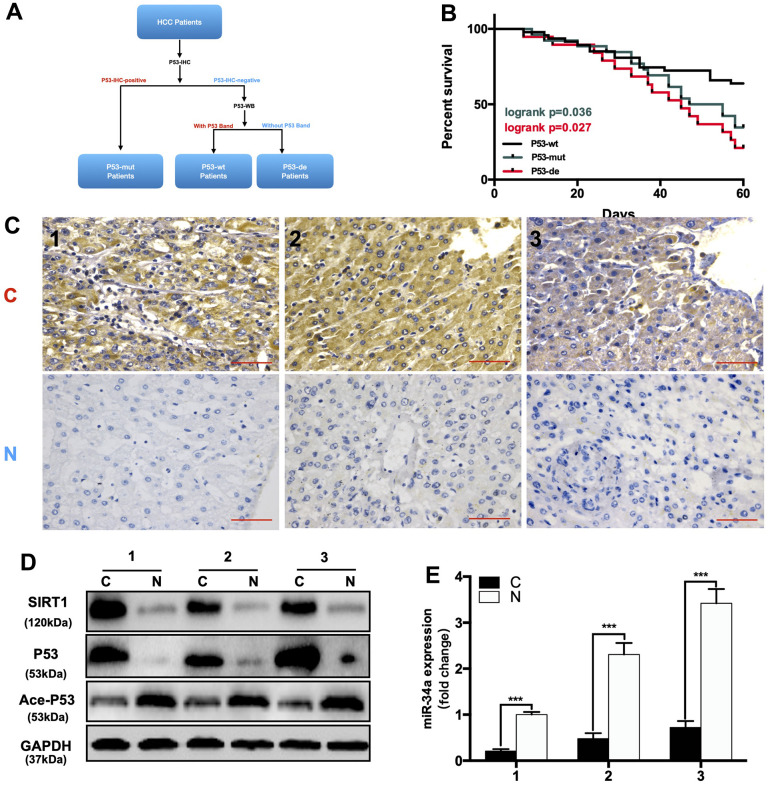
**The expression of the P53/miR-34a/SIRT1 positive feedback loop in hepatocellular carcinoma patients.** (**A**) A brief diagram of how 92 HCC patients were divided. (**B**) The 5-year survival rate of HCC patients from different groups. (p = 0.036, Green: P53-mutated vs P53-wild-type patients. p = 0.027, Red: P53-deficient vs P53-wild-type patients, log-rank test). (**C**) Representative images of P53 staining in P53-mutated patients. (**D**) Protein expression of the P53/miR-34a/SIRT1 positive feedback loop in P53-mutated patient tumor tissue. (**E**) Quantification of hepatic miR-34a expression in P53-mutated patient tumor tissue.

**Figure 6:**
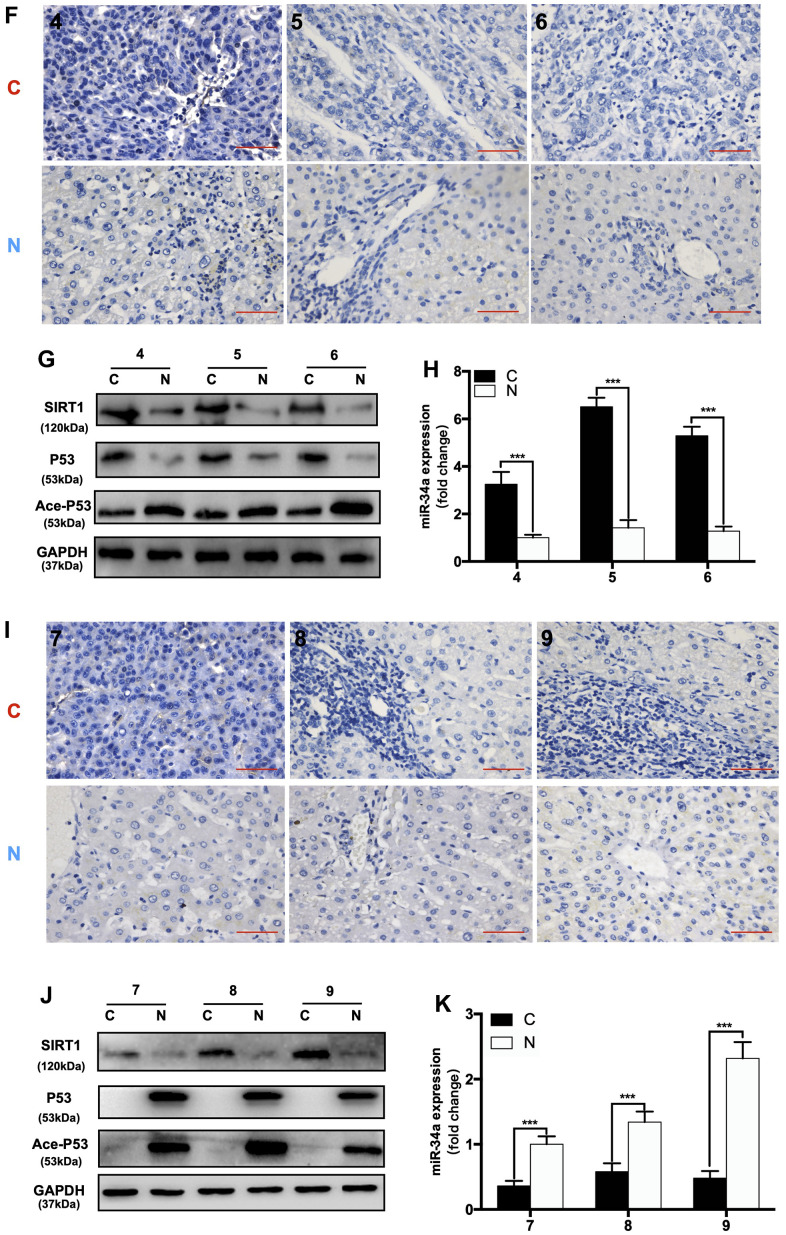
**The expression of the P53/miR-34a/SIRT1 positive feedback loop in hepatocellular carcinoma patients.** (**F**) Representative images of P53 staining in P53-wild-type patients. (**G**) Protein expression of the P53/miR-34a/SIRT1 positive feedback loop in P53-wild-type patient tumor tissue. (**H**) Quantification of hepatic miR-34a expression in P53-wild-type patient tumor tissue. (**I**) Representative images of P53 staining in P53-deficient patients. (**J**) Protein expression of the P53/miR-34a/SIRT1 positive feedback loop in P53-deficient patient tumor tissue. (**K**) Quantification of hepatic miR-34a expression in P53-deficient patient tumor tissue. C: liver cancer tissue N: adjacent normal liver tissue (***, p<0.001).

**Table 1:** Relationship between P53 expression and clinicopathological features of HCC patients.

**Characteristics**	**P53-wt**	**P53-mut**	**P53-de**	**P-Value^a^**	**P-Value^b^**
**Age**					
≤50	19	12	9		
>50	28	14	10	0.8051	0.7838
**Gender**					
Male	23	14	10		
Female	24	12	9	0.8079	1
**Tumor Size**					
≤5cm	33	10	6		
>5cm	14	16	13	**0.0127**	**0.0057**
**Distant Metastasis**					
Absent	38	12	7		
Present	11	14	12	**0.0096**	**0.0034**
**Lymphatic Metastasis**					
Absent	20	13	11		
Present	27	13	8	0.6262	0.2881

P < 0.05 was considered statistically significant (bold values*).

^a^Comparing between P53-wt and P53-mut group using chi-square test.

^b^Comparing between P53-wt and P53-de group using chi-square test.

## DISCUSSION

The liver is extremely vulnerable to physical and chemical damage. LR protects the liver by assisting it in recovering from such injury. However, the precise processes behind the termination stage of LR following PH remain unknown. In our investigation, we found that the P53/miR-34a/SIRT1 positive feedback loop was considerably active in the late stage of LR. Moreover, overexpression of P53 increased apoptosis of hepatocytes during LR and ended LR beforehand, while knock-down of miR-34a abolished P53/miR-34a/SIRT1 positive feedback loop during LR and suppressed LR termination (Figures 2, 3). Here, we suggest a novel mechanism that governs the termination of LR, which further the knowledge of its underlying regulatory mechanism. The P53/miR-34a/SIRT1 positive feedback loop has been implicated in preventing cell growth and triggering cell death in a variety of cell lines and diseases, our research will provide new insights into the study of P53/miR-34a/SIRT1 in both LR and tumorigenesis.

Yamakuchi and his colleagues overexpressed miR-34a and found a lower SIRT1 level, resulting in higher acetylated P53 level and enhanced levels of P53 [9]. It has been shown that the P53/miR-34a/SIRT1 positive feedback loop is activated according to severity of human non-alcoholic fatty liver disease (NAFLD), and several antitumor drugs could activate the P53/miR-34a/SIRT1 positive feedback loop in cancer cell lines to induce the apoptosis of cancer cells [26, 27]. Duarte M. S. Ferreira et al. demonstrated that P53/miR-34a/SIRT1 activated by JNK1/c-Jun contributed to cell death caused by deoxycholic acid in rats [28]. In our study, the P53/miR-34a/SIRT1 positive feedback loop was activated in the late stage of LR, and its proapoptotic and antiproliferative effects could be necessary to terminate LR. ALT and AST levels in mice serum after PH did not recover to normal in mice overexpressing P53, which might be due to liver regeneration in mice overexpressing P53 being terminated earlier than under normal conditions. Overexpression of P53 intensified the effect of the P53/miR-34a/SIRT1 positive feedback loop in the termination stage of LR at 7 days after PH (Figure 2K). Our findings suggest that the P53/miR-34a/SIRT1 positive feedback loop is activated during the termination stage of LR rather than the initiation or proliferation stage. Although there was an increase in P53, it failed to stimulate the P53/miR-34a/SIRT1 positive feedback loop in the early phase of LR (Figure 1A). Thus, there might be a certain mechanism that controls the activation of the P53/miR-34a/SIRT1 positive feedback loop. Meanwhile, knocked-down of miR-34a suppressed the P53/miR-34a/SIRT1 positive feedback loop and its effect (Figure 3G, 3H). LR became infinitely and could not be terminated during the first 14 days after PH (Figure 3B). These data suggest P53/miR-34a/SIRT1 positive feedback loop is essential for LR termination.

In our study, we found that total BA levels were in the early phase of LR and lower in the termination stage of LR, with a gradual increase in the proportion of T-β-MCA over total BA (Figure 4B, 4C). Sama I. Sayin et al. identified T-β-MCA as a FXR antagonista, which suppressed the FXR/SHP pathway [22]. Our study showed that T-β-MCA suppressed the FXR/SHP signaling pathway and amplified the proapoptotic effects of P53-induced activation of the P53/miR-34a/SIRT1 positive feedback loop both *in vitro* and *in vivo*.

TP53 mutations are commonly found in HCC patients. Previous research showed that TP53 had the highest prevalence of protein-altering mutations in HCC [29, 30]. Several studies have shown that tumor drugs can suppress the proliferation of liver cancer cells by activating the P53/miR-34a/SIRT1 positive feedback loop [27, 31]. In our study, we show that the P53/miR-34a/SIRT1 positive feedback loop is deficient P53-mut and P53-de patients. Thus, deficiency in the P53/miR-34a/SIRT1 positive feedback loop induced by the mutation or loss of P53 might be the cause of HCC tumorigenesis and development.

## CONCLUSIONS

In summary, a P53/miR-34a/SIRT1 positive feedback loop exists and is activated in the termination of LR. The effect of the P53/miR-34a/SIRT1 positive feedback loop is regulated by increased or decreased level of T-β-MCA via the FXR/SHP pathway, and the mechanism is summarized in the diagram (Graphical Abstract). These findings may provide new insight into the treatment of chronic liver diseases, liver transplantation, and liver malignancy, especially for HCC patients with different P53 expression.

## Supplementary Material

Supplementary Figures

Supplementary Tables
