# Supplementary material for: P53/miR-34a/SIRT1 positive feedback loop regulates the termination of liver regeneration
Source: Aging (Albany NY). 2023 Mar 28;15(6):1859–77. doi: 10.18632/aging.203920 (PMC10085612; doi:10.18632/aging.203920)
Supplement: Supplementary Figures [file aging-15-203920-s001.pdf]

SUPPLEMENTARY FIGURES

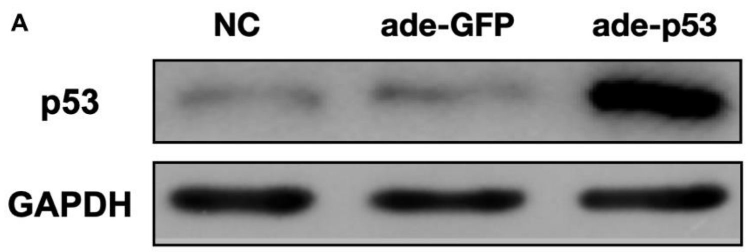

Supplementary Figure 1. Transfection efficiency of adenovirus *in vivo*. Mice were transfected with either Ade-GFP (as the control) or Ade-P53 by tail vein injection five days before the surgeries. Expression of pP53 in the liver tissues analyzed by WB.

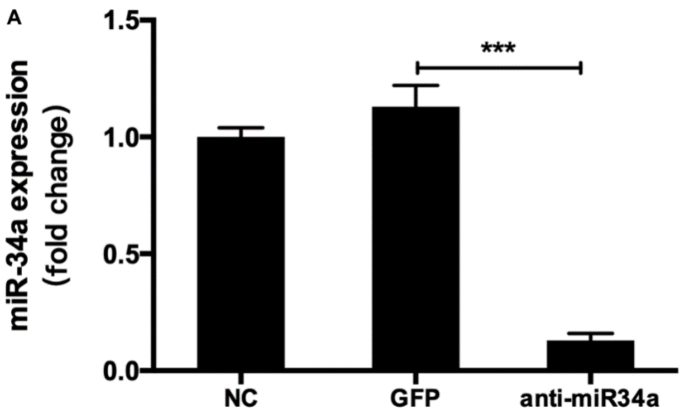

Supplementary Figure 2. Transfection efficiency of AAV8 *in vivo*. Mice were transfected with either AAV8-GFP (as the control) or AAV8-anti-miR-34a by tail vein injection two weeks before the surgeries. Expression of miR-34a in the liver tissues analyzed by qPCR. (values are means ±SD, ***$P < 0.05$).

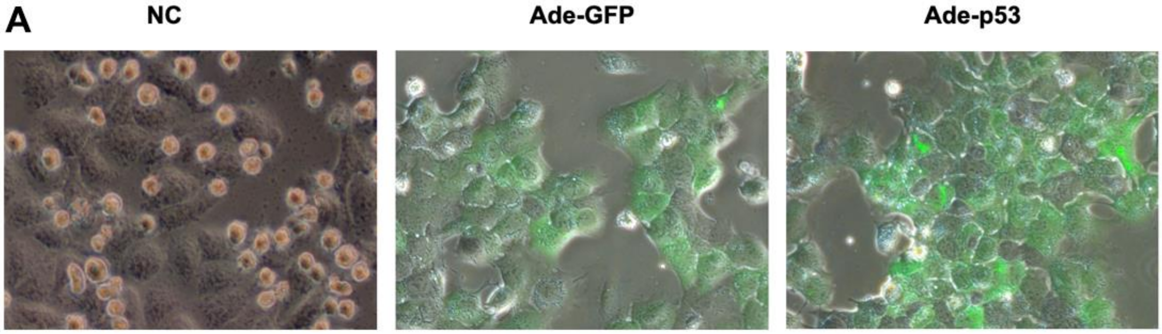

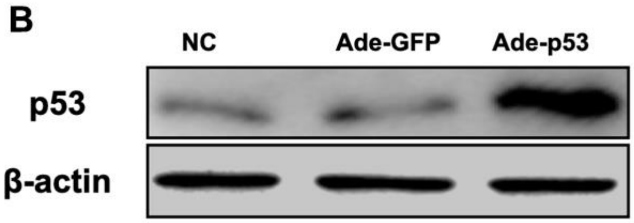

Supplementary Figure 3. Transfection efficiency of hepatocytes *in vitro.* Transfection efficiency of hepatocytes *in vitro.* Primary hepatocytes were transfected by adenovirus according to the manufacturer's instructions, and cells transfected with GFP were used as control. (**A**) Hepatocytes were observed under light and fluorescence microscope after transfection. (**B**) Expression of P53 in the hepatocytes analyzed by western blot.
