# Supplementary material for: P53/miR-34a/SIRT1 positive feedback loop regulates the termination of liver regeneration
Source: Aging (Albany NY). 2023 Mar 28;15(6):1859–77. doi: 10.18632/aging.203920 (PMC10085612; doi:10.18632/aging.203920)
Supplement: Supplementary Tables [file aging-15-203920-s002.pdf]

SUPPLEMENTARY TABLES

Supplementary Table 1. List of BAs assessed by UHPLC.

T-α-MCA	Tauro-alpha-Muricholic acid
T-β-MCA	Tauro-beta-Muricholic acid
THCA	Taurohyocholic acid
TUDCA	Tauroursodeoxycholic acid
TCA	Taurocholic acid
GCA	Glycocholic acid
ω-MCA	Omega-Muricholic acid
α-MCA	Alpha-Muricholic acid
β-MCA	Beta-Muricholic acid
HCA	Hyocholic acid
TDCA	Taurodeoxycholic acid
CA	Cholic acid
UDCA	Ursodeoxycholic acid
HDCA	Hyodeoxycholic acid
nutriCA	Nutri cholic acid
12-ketoDCA	12-keto deoxycholic acid
TLCA	Taurolithocholic acid
CDCA	Chenodeoxycholic acid
DCA	Deoxycholic acid
GLCA	Glycolithocholic acid
iso-DCA	Iso-lithocholic acid
LCA	Lithocholic acid

Supplementary Table 2. List of primary antibody and concentration used in WB.

Target protein	Catalog number	Company	Concentration
P53	Ab26	Abcam, Inc.	1:1000
Ace-P53	2570S	CST	1:1000
SIRT1	8469S	CST	1:1000
cleaved Caspase3	Ab231289	Abcam, Inc.	1:1000
P21	Ab188224	Abcam, Inc.	1:1000
Bax	Ab32503	Abcam, Inc.	1:1000
NR1H4 (FXR)	Ab187735	Abcam, Inc.	1:1000
NR0B2 (SHP)	Ab186874	Abcam, Inc.	1:1000
